# La Grippe

**Published:** 1890-03

**Authors:** Samuel Swan

**Affiliations:** New York


### LA GRIPPE.

SAMUEL SWAN, M. D., NEW YORK.

The wide prevalence of la grippe, and its cause, have been commented on extensively, but with no very definite result. Certain atmospheric conditions may lead to a clue as to cause. Some years since, it is reported, the mercury in St. Petersburg rose thirty-two degrees in one night, and the next day there were forty thousand cases of the grippe. Did the sudden rise in temperature come from the lessening of ozone in the atmosphere? for it was said to be below normal the next day. If investigations have been made in this direction they have not appeared in print, but it would be well to fill a chamber, where there were persons sick of the disease, with pure oxygen and ascertain the effect. There does not appear to be any symptom of the disease itself, to make it fatal, and cases left to expectant treatment have recovered; but the parties were strong, healthy persons when attacked. Now there is no reason for a strong, healthy person dying from the grippe, but such *have* died and I think the cause lies in the great quantity of that deadly poison, *Quinine*, which is given indiscriminately to all. The grippe is a disease of a depressing nature, and, when added to that, we have the terribly depressing after-effect of Quinine—it is more than the vital force can react against and the croupous bronchitis is followed by croupous pneumonia, and death closes the scene. But this is not all. The insanities, the suicides, the murders are greatly due to this deadly poison. Hahnemann made a careful investigation of its effects on himself and his friends, for it was from this drug, the Cinchona, that he made his discovery of the law, *Similia similibus curantur*. I will quote from his provings some of the mental symptoms, and the alternate conditions of exaltation and depression are recognized by every observant physician:

"Intolerable anxiety, he jumps out of bed, and wants to kill himself, and, nevertheless, dreads to approach the window or the knife. He tosses about the bed, beside himself, and in despair; inconsolable, distressing, moaning, and screaming; taciturn; obstinate silence; disobedience; want of docility. Ill humor increased by caresses. He despiseth everything; dissatisfaction; he thinks he is unhappy, and imagines he is tormented and teased by everybody. He is vexed and gets easily angry.

Anger increasing to the most violent wrath; he could have stabbed one. Inclines to feel angry, and seeks opportunities for it; afterward quarrelsome and disposed to grieve and reproach others. Congestion of the brain, and abolishes the cerebral functions. It causes deafness, and serious inflammation of the internal ear. Blindness, ischæmia of the retina; neuritis. It produces stupor, delirium, and convulsions. Disturbance, or rather emptiness of the mind—insanity, excitement. Feeling of impending evil in the afternoon; fretfulness; anger after sleep, even about a draught on the legs. Apathy, indolence, disinclination for mental labor. Thought difficult when writing. Memory 'muddled'—mistakes in writing 'left' for 'right' and *vice versa.* Loss of power to name substances; mistakes in adding figures; perception of quantities impaired; vacancy of ideas."

Quinine has a specific action on the spinal marrow and spinal nerves. Its first effect is to excite the nervous action, which is followed by a depression of the vital functions, and an increase of sensibility—and the depression is more profound than the previous exaltation. Characteristic indications are general languor, sudden sinking of strength, trembling of the limbs, nervous twitchings, convulsions; violent headache in the vertex, causing great anguish and delirium. It is not my intention to give more than an outline of the effects, but I quote from the respiratory organs to show why it produces pneumonia, and why people, who have habitually taken Quinine, generally die when attacked by pneumonia.

"Hoarseness from mucus in the larynx. Whistling, whizzing, rattling in the trachea and larynx. Tracheitis—catarrh of the trachea and bronchi. Cough from constant irritation in the throat, as from the vapor of sulphur, without expectoration. Nocturnal suffocating cough, like whooping cough, with intense pain. Violent cough after every meal. Cough with expectoration of blood-streaked mucus. Cough with difficult expectoration of clear tenacious mucus, with painful concussion of the scapula. Hemorrhage from the lungs. Cough with purulent expectoration. Adynamic preliminary phthisis, with profuse purulent discharge, loss of strength, evening fever and night sweats."

Why sudden deaths occur, may be inferred from the following heart symptoms: "Faintness; fell suddenly in the street; fell to the earth senseless; rush of blood to the head and face, which was red and hot, with coldness of the hands; violent palpitation of the heart; sensation as if the heart had stopped, pulse cannot be felt; heart ache; heart failure; melancholy feeling about the heart, with desire to take a deep inspiration."

It will be observed how few stimulating symptoms there are compared with the depressing ones. Business men, brokers and lawyers, men engaged in business that causes "brain fag," generally keep a box of Quinine pills in their pocket, and when they feel themselves "letting down" they take a pill as a "pick-me-up." But should they have pneumonia, and they are apt to be attacked with it suddenly, they will surely die; an eminent physician, lately deceased, than whom there was never a more careful observer, gave it as *his* belief, the result of many years' experience, and my own observation has confirmed its truth. I think if the truth could be known, hardly a person has died of the grippe that has not taken Quinine. But not all have died of pneumonia. The violent, unbearable headache, principally in the top of the head, is followed by coma, varied by violent delirium, stupor, and cerebral apoplexy preceded by the redness of the face closes the scene, and this condition is caused by Quinine.

It was reported in the papers that on Monday, the 6th of January, there were eighty-two corpses in the morgue, and that they were all the bodies of suicides. The great depression and hopelessness resulting from Quinine with the desire to kill, would account for many or most of these suicides.

All of the symptoms mentioned above are liable to appear when massive doses of Quinine are given—but from want of knowledge, physicians declare Quinine to be harmless, and give from five to fifty grains at a dose; and the result of too much is delirium and death. Antipyrine is now being proven, and no doubt we shall find another deadly poison, which is given without knowledge and without judgment. If physicians were examined as to their knowledge of *drug action*, the surprise would be that they ever dared recklessly to give such poisons at all.

Most of the above symptoms were obtained by the proving of Cinchona officinalis, or Peruvian bark, while Quinine is cinchona mixed with sulphur, making Chinium sulphuricum; but the addition of sulphur only intensifies its action, as there is no greater depresser than sulphur.

If this paper will "call a halt" in the use of Quinine, the object of the writer will have been attained.

